# Are native and non‐native pollinator friendly plants equally valuable for native wild bee communities?

**DOI:** 10.1002/ece3.6826

**Published:** 2020-10-13

**Authors:** Nicola Seitz, Dennis vanEngelsdorp, Sara D. Leonhardt

**Affiliations:** ^1^ Department of Animal Ecology and Tropical Biology University of Würzburg Würzburg Germany; ^2^ Department of Entomology University of Maryland College Park MD USA; ^3^ Present address: Department of Ecology & Ecosystem Management Technical University of Munich Freising Germany

**Keywords:** bee conservation, common garden experiment, exotic plants, non‐native plants, plant–bee visitation networks, pollinator friendly plants, wild bees

## Abstract

Bees rely on floral pollen and nectar for food. Therefore, pollinator friendly plantings are often used to enrich habitats in bee conservation efforts. As part of these plantings, non‐native plants may provide valuable floral resources, but their effects on native bee communities have not been assessed in direct comparison with native pollinator friendly plantings. In this study, we performed a common garden experiment by seeding mixes of 20 native and 20 non‐native pollinator friendly plant species at separate neighboring plots at three sites in Maryland, USA, and recorded flower visitors for 2 years. A total of 3,744 bees (120 species) were collected. Bee abundance and species richness were either similar across plant types (midseason and for abundance also late season) or lower at native than at non‐native plots (early season and for richness also late season). The overall bee community composition differed significantly between native and non‐native plots, with 11 and 23 bee species being found exclusively at one plot type or the other, respectively. Additionally, some species were more abundant at native plant plots, while others were more abundant at non‐natives. Native plants hosted more specialized plant–bee visitation networks than non‐native plants. Three species out of the five most abundant bee species were more specialized when foraging on native plants than on non‐native plants. Overall, visitation networks were more specialized in the early season than in late seasons. Our findings suggest that non‐native plants can benefit native pollinators, but may alter foraging patterns, bee community assemblage, and bee–plant network structures.

## Graphical abstract

We show that non‐native plants are similarly or even more attractive to native bees than native plants. However, foraging patterns, bee community assemblage, and bee‐plant network structures were different with lower levels of specialization at non‐native plants. Thus, adding non‐native plants to native pollinator friendly plantings can be beneficial to wild bees, but should be confined to already disturbed human‐dominated landscapes to exclude potentially negative effects on native plant communities.

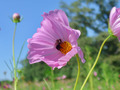

## 1 INTRODUCTION

Bees are important pollinators worldwide and largely rely on pollen and nectar of plants as main food resources (Carvell et al., 2006; Michener, 2007; Vaudo et al., 2015). Reductions in plant abundance and diversity as a consequence of land use change are considered one of the reasons for the currently observed decline in bee populations worldwide (Baude et al., 2016; Kennedy et al., 2013; Robinson & Sutherland, 2002; Winfree et al., 2011). As a consequence, providing adequate and sufficient floral resources may be a key measure for supporting wild bee populations (Vanbergen & Initiative, 2013). To provide food resources for bees, many seed companies, botanical associations, and other interest groups have compiled and promoted “pollinator friendly” plant lists and/or seed mixes. The nectar and/or pollen from these mixes of plants are believed attractive to pollinators, but this assumption has not been extensively tested (Ratnieks & Garbuzov, 2014). Hicks et al. (2016) showed that nectar and pollen quantity and quality varied among 23 pollinator friendly plants, but they did not assess bee visitation, while Garbuzov and Ratnieks (2014) found highly fluctuating bee visitation rates among 32 UK garden plants. Therefore, the attractiveness of pollinator friendly plants for bees deserves further exploration.

Many commercially available pollinator friendly plant mixes often include both native and non‐native plants (Fowler, 2016; Morandin & Kremen, 2013). Selecting plants for pollinators based on the quality or quantity of floral rewards, rather than their native range, may benefit bees and support conservation efforts. However, non‐native plants may adversely affect native bee communities by providing floral resources of inappropriate nutritional value or by benefiting generalist over specialist bee species. The (long‐term) effect of non‐native plants on bee communities warrants further investigation (Garibaldi et al., 2013; Klein et al., 2003; Morandin & Winston, 2005; Ollerton et al., 2011).

**Figure 1:**
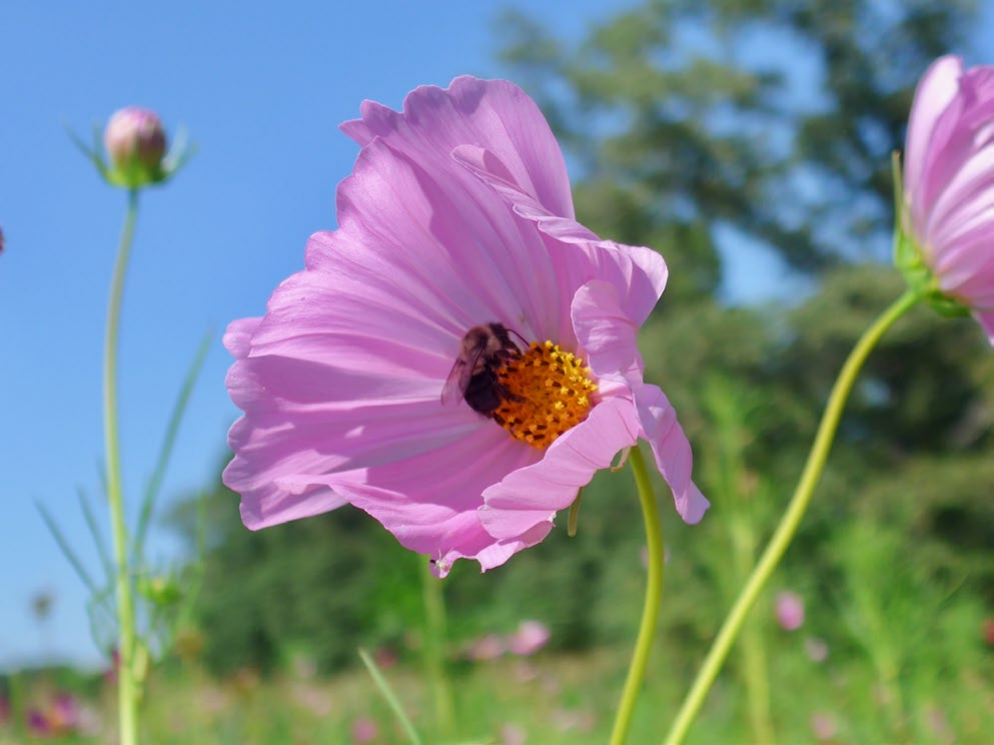
Native bumble bee (*Bombus impatiens/bimaculatus*) visiting a non‐native cosmos plant (*Cosmos bipinnatus*), Maryland, USA

Flowers of non‐native plants are foraged on by native bees (Drossart et al., 2017; Tepedino et al., 2009; Williams et al., 2011; Figure 1), but whether they prefer native or non‐native plants is still unclear. While Williams et al. (2011), Nienhuis et al. (2009), and Morales and Aizen (2006) found no differences in bee visitation between native and non‐native plants, other studies report either higher bee abundances (Bartomeus et al., 2008; Gibson et al., 2013; Lopezaraiza‐Mikel et al., 2007; Matteson & Langellotto, 2010; Vilà et al., 2009) or lower bee abundances for non‐native plants (Chrobock et al., 2013; Menz et al., 2011; Morandin & Kremen, 2013; Moroń et al., 2009; Pardee & Philpott, 2014). Similarly, studies found bee species richness either higher (Bartomeus et al., 2008; Lopezaraiza‐Mikel et al., 2007; Vilà et al., 2009), lower (Chrobock, et al., 2013; Morandin & Kremen, 2013), or the same (Pardee & Philpott, 2014) on non‐native plants versus native plants. The majority of this research has been conducted in Europe and the United States, while studies from the southern hemisphere are still scarce (but see Morales and Aizen (2006) and Gibson et al. (2013) for examples from Argentina and South Africa) despite the often negative effects of non‐native (invasive) plant species on native flora and fauna (Bellard et al., 2017; Van Kleunen et al., 2015). Most of these studies focused on invasive non‐native plants instead of directed pollinator friendly plantings and were observational, rather than the result of hypothesis‐driven field trials. An exception was the study by Morandin and Kremen (2013) who analyzed hedgerows planted for natural habitat restoration along agricultural fields in the United States. However, in their study, native and non‐native plants were mixed in the same treatments and were not explicitly pollinator friendly. To date, no study has experimentally assessed whether flowers of planted native and non‐native pollinator friendly seed mixes differently affect pollinator communities.

Several studies document that the visitors of non‐native flowers are less specialized than those visiting native flowers (Grass et al., 2013; Lopezaraiza‐Mikel et al., 2007; Memmott & Waser, 2002; Schweiger et al., 2010; Stout & Morales, 2009), suggesting that non‐native plants support generalist bees and disproportionally affect specialist bee species, which are overall more sensitive to land use change than generalists (Winfree et al., 2011). This can alter the community composition and the structure of the bee visitation network (Bartomeus et al., 2008; Olesen et al., 2002; Vanbergen et al., 2018), while overall bee abundance or species richness may not be affected.

In this 2‐year study, we experimentally tested how flowering plants grown from seed mixes composed of either native or non‐native seeds affected the abundance, species richness, and community structure of bees. We also investigated the specialization of visitation networks and the specialization of individual bee species. As both native and non‐native seed mixes were comprised of bee‐friendly plant species, we expected similar bee abundances and species richness for native and non‐native plantings. Furthermore, we expected bees to forage in a more specialized manner on native than non‐native plants. Our study is the first to experimentally compare native versus non‐native pollinator friendly plant communities planted in plots of the same size in a common garden experiment.

## 2 METHODS

### 2.1 Study system

**Figure 2:**
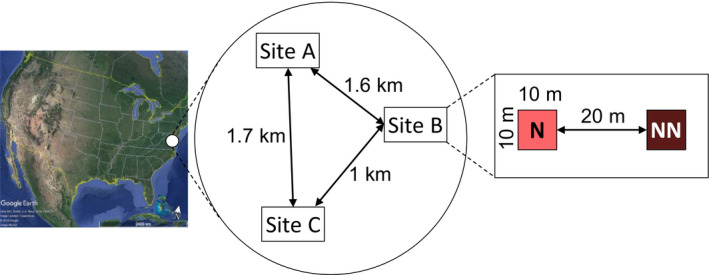
Overview of the three experimental sites (A, B, and C) at the Beltsville research farm. At each site, a native plant plot (N) and a non‐native plant plot (NN) were established in 2016. Map data: Google Earth, US Dept of State Geographer, Image Landsat/Copernicus, Data SIO, NOAA, U.S. Navy, NGA, GEBCO

**Figure 3:**
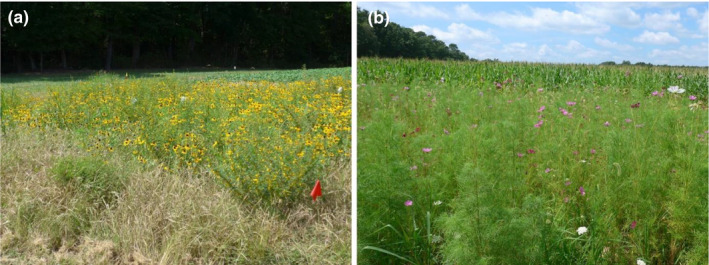
Examples of experimental plantings at site B with (a) native and (b) non‐native seed mixes

The study took place at the Beltsville research farm of the University of Maryland, USA. The Beltsville facility spans 116 ha in a rural landscape with forest fragments, creeks, ponds, and agricultural fields. At the research farm, we established three experimental sites which were 1–1.7 km apart from each other (Figure 2). Each site had two plots: one sown with a native pollinator friendly seed mix and one sown with a non‐native pollinator friendly seed mix (Figures 2 and 3). Plots were 10 × 10 m in size, and the distance between the two treatment plots was 20 m. The central coordinates for the three sites were 39.025705, −76.842232 (site A); 39.018170, −76.821249 (site B); and 39.007196, −76.820480 (site C).

Both the native and non‐native seed mixes contained 20 different flower species and two grass species each (Tables 1 and 2). Grasses are typically added to pollinator friendly meadows as they also provide nesting or overwintering sites for insects and can protect wildflowers by preventing soil erosion (Lee‐Mäder et al., 2013). The plants in the seed mixes were chosen to fulfill the following criteria: (a) pollinator friendliness as assessed through consulting existing databases on pollinator friendly plants (i.e., by The Xerces Society, Pollinator Partnership, USDA, Royal Horticultural Society, Ernst Conservation Seeds); (b) complementary flowering periods spanning the whole season of bee activity; (c) an even distribution of flower colors; (d) a mix of different plant families; (e) mostly perennials (12–15 species) with a few annuals/biennials (5–8 species) to facilitate establishment in the first year; (f) adaptation to medium to dry and sandy soil; and (g) availability from seed retailers.

**Table 1:** List of native plants used in the native plant seed mix with respective flowering periods

Plant species	Year	Apr	May	Jun	Jul	Aug	Sep	Oct
*Asclepias tuberosa*	2016							
	2017							
*Baptisia australis*	2016							
	2017							
*Bidens aristosa*	2016							
	2017							
*Chamaecrista fasciculata*	2016							
	2017							
*Chamaecrista nictitans*	2016							
	2017							
*Eupatorium perfoliatum*	2016							
	2017							
*Helenium flexuosum*	2016							
	2017							
*Lespedeza virginica*	2016							
	2017							
*Liatris pilosa*	2016							
	2017							
*Lupinus perennis*	2016							
	2017							
*Monarda punctata*	2016							
	2017			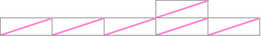				
*Penstemon laevigatus*	2016							
	2017							
*Pycnanthemum tenuifolium*	2016							
	2017							
*Rudbeckia hirta*	2016							
	2017							
*Rudbeckia triloba*	2016							
	2017							
*Sisyrinchium angustifolium*	2016							
	2017							
*Solidago odora/nemoralis*	2016							
	2017							
*Symphyotrichum laeve*	2016							
	2017							
*Tradescantia virginiana*	2016							
	2017							
*Verbena hastata*	2016							
	2017							

Note

The months of flowering are indicated with bars in colors corresponding to the color of the flowers. In 2016, 10 out of 20 native plants were flowering. In 2017, 17 native plants were flowering. This seed mix additionally contained the two grass species *Elymus virginicus* and *Schizachyrium scoparium*.

**Table 2:** List of non‐native plants used in the non‐native plant seed mix with respective flowering periods

Plant species	Year	Apr	May	Jun	Jul	Aug	Sep	Oct
*Achillea millefolium*	2016							
	2017							
*Agastache foeniculum*	2016							
	2017							
*Calendula officinalis*	2016							
	2017							
*Cichorium intybus*	2016							
	2017							
*Coronilla varia*	2016							
	2017							
*Cosmos bipinnatus*	2016							
	2017							
*Daucus carota*	2016							
	2017							
*Leucanthemum maximum*	2016							
	2017							
*Leucanthemum vulgare*	2016							
	2017							
*Linum perenne*	2016							
	2017							
*Lobularia maritima*	2016							
	2017							
*Lotus corniculatus*	2016							
	2017							
*Melilotus officinalis*	2016							
	2017							
*Origanum vulgare*	2016							
	2017							
*Papaver rhoeas*	2016							
	2017							
*Salvia officinalis*	2016							
	2017							
*Trifolium incarnatum*	2016							
	2017							
*Trifolium pratense*	2016							
	2017							
*Trifolium repens*	2016							
	2017							
*Viola cornuta*	2016							
	2017							

Note

The months of flowering are indicated with bars in colors corresponding to the color of the flowers. In 2016, eight out of 20 non‐native plants were flowering. In 2017, 17 non‐native plants were flowering. This seed mix additionally contained the two grass species *Dactylis glomeratus* and *Eragrostis curvula*.

The plots were seeded in April 2016 and reseeded in March 2017. In 2017, *Solidago odora* seeds were not available and were replaced with *Solidago nemoralis* seeds. Throughout both years, the plots were continuously hand‐weeded.

### 2.2 Bee sampling

Bees were sampled by hand netting and with pan traps between April and October in 2016 and 2017. Sampling took place approximately every 2 weeks (13.3 ± 5.7 days (mean ± *SD*) between consecutive sampling events) on rain‐free and (mostly) sunny days. Each year, hand netting started as soon as the first plants started to bloom (resulting in different starting points for native and non‐native plots) and was performed during 30‐min random walks through plots: All bees observed to touch reproductive flower parts were captured from flowers with a ziplock plastic bag. Nectar‐ or pollen‐gathering behavior was not distinguished. We stored bees in 70% ethanol for identification. All plots were sampled within 1 day between 9:00 and 18:00 in alternating random order. Hand netting was always performed by the same person (N. Seitz). Note that *Cichorium intybus* closed its flowers very early in the day, which is why these plants could not be observed later in the day. We therefore conducted additional sampling of *C. intybus* in the morning of the next day (2 August 2017 and 25 August 2017). Furthermore, sampling on 1 August 2017 was interrupted by rain and therefore continued the next day. Over 2 years, 21 hand netting events were conducted across sites. In 2016, non‐native plots were sampled eight times and native plots five times due to a later onset of flowering at native plots. In 2017, non‐native plots were sampled 13 times and native plots 11 times at site B and 10 times at sites A and C due to a later onset of flowering at these sites. We at least partly accounted for these differences in flowering time by calculating bee numbers per sampling event (see 2.3 Statistics for details).

On each day of hand netting, bees were also sampled with pan traps (except for 15 July 2016). Additionally, we sampled bees with pan traps once in April 2016 and once in May 2016 before plants started flowering. We followed guidelines by Droege (2015) for pan trap sampling. We placed twelve pan traps (3.5 oz) filled with soapy water and colored in fluorescent blue, yellow, and white along plot edges in the morning, before the hand netting started. We recollected the traps in the evening, after hand netting was finished. When recollecting traps, we drained the soap water with paper–nylon mesh paint strainers (190 μm) and stored the specimens separated by plot in 70% ethanol. The sampling days of pan trap sampling were identical for all plots. Overall, we obtained 9 and 13 samples per plot or 54 or 78 samples in total, in 2016 and 2017, respectively.

On all sampling days, we recorded the weather (sunny/cloudy), obtained current temperature, and predicted maximal temperature and wind speed from http://weather.com. In 2017, we estimated the floral cover (proportion of ground with flowering vegetation) across the entire 10 × 10 m plot on sampling days.

Final species determination was done by Sam Droege at the USGS Patuxent Wildlife Research Center. Species and sampling information of all bees were made publicly available at www.discoverlife.org. Note that bee individuals of *Halictus poeyi* and *Halictus ligatus* were indistinguishable and therefore placed in one group (i.e., *Halictus poeyi/ligatus*) as were *Hylaeus affinis* and *Hylaeus modestus*.

### 2.3 Statistics

All statistical analyses were performed with R version R 3.5.1 (R Core Team, 2018) in RStudio version 1.1.456 (RStudio Team, 2016). Data were collected over a period of 4–6 months per year corresponding to the entire flowering period of experimental plants. The data set therefore comprised bee and plant species that did not necessarily co‐occur due to different phenologies. To restrict network analyses to co‐occurring species, we followed the approach by Kantsa et al. (2017) of composing several phenologically matched networks. We differentiated between three partly overlapping seasons, that is, spring to early summer (April 1–July 15), midsummer (June 1–August 31), and late summer to fall (July 16–October 3). For each season, data were pooled across years. Midsummer overlapped with the early and late seasons in order to smoothly incorporate the phase of transition from early to late season, capturing the late species of the early season and the early species of the late season. It is nevertheless important to treat midsummer as a distinct season, as many studies often focus exclusively on this time period (Chrobock, et al., 2013; Cusser & Goodell, 2013; Fründ et al., 2010; Hegland et al., 2010).

We visualized and analyzed plant–bee visitation networks with the “bipartite” package (functions “plotweb,” “networklevel,” and “specieslevel” (Dormann et al., 2008)). Species that could be identified to genus level only were included in networks if no other species of the same genus was present (i.e., *Melissodes* and *Sphecodes)*. The H2′ index within the function “networklevel” was used to compare the community‐level specialization of each network (Blüthgen et al., 2006). At the species level, we used the *d*′ index of the function “specieslevel” to compare the specializations in plant visitation of single bee species within a network (Blüthgen et al., 2006). We assessed species specificity (*d*′) of the five most abundant bee species and only included those *d*′ values for these species that were based on more than two observations per network.

We analyzed differences in bee species richness per sampling event, abundance per sampling event, network specificity (H2′) per season, and species specificity (*d*′) per season between native and non‐native plots using linear mixed‐effect models (LMM; “lmer” function, “lme4” package (Bates et al., 2015)). Data were log‐transformed where necessary (i.e., bee species richness and abundance) in order to achieve a normal distribution (as visually assessed with histograms and tested with Shapiro tests). We calculated models for species richness and abundance (per sampling event) for each season and included plant nativity as a fixed effect and site and Julian date as random effects. As floral variables were measured in 2017 only, we did not include them in our main models. However, we composed additional models for the data of 2017 which included floral abundance and floral richness as fixed effects to test for their effect on bee diversity and abundance (see Table S3).

Due to the earlier flowering of non‐native plants resulting in a higher number of sampling events at non‐native plots, we based our comparisons of bee abundance and richness on visitations per sampling event. To additionally account for the uneven sampling, we performed supplementary analyses for spring/early summer (the only season were sampling days differed) which were restricted to those sampling events where plants of both types were flowering.

For network and species specificity, we obtained always one H2′ value and one *d*′ value (per species) for each of the 18 networks (i.e., each native and non‐native plot at each of the three sites for each of the three seasons pooled across years). Our models for network and species specificity included plant nativity and season as fixed effects and site as a random effect. To ensure that trends in network specificity were not heavily influenced by the behavior of the non‐native and domesticated honeybee (*Apis mellifera*), we performed additional network analyses excluding this species.

Significance of fixed effects (plant nativity and season) was assessed with the “ANOVA” command of the “car” package. Multiple comparison of means for differences between seasons was analyzed using Tukey's post hoc tests (“glht” function, “multcomp” package (Hothorn et al., 2008)). To finally assess the overall variance explained by models, we calculated *R^2^*‐values with the pseudo‐R‐squared (“r.squaredGLMM”) function of the “MuMIn” package (Bartoń, 2018).

To analyze bee community composition, we visualized data using non‐metric multidimensional scaling (NMDS; function “metaMDS,” “vegan” package) with a Bray–Curtis distance matrix based on the abundances of different bee species (Oksanen et al., 2018). Differences in bee community composition between native and non‐native plots were then assessed using a permutational multivariate analysis of variance (PERMANOVA; “adonis” function in the “vegan” package; 100 000 permutations) also based on Bray–Curtis distances between abundances of bee species (“vegdist” function, “vegan” package (Oksanen et al., 2018)). As only bees were included in this analysis and not their temporally changing interactions with plants, we only included data of the early and late seasons here and excluded the midseason to avoid pseudoreplication.

Network analyses as well as statistical analyses on differences of bee abundance, species richness, Shannon diversity, and bee community composition between native and non‐native plots were based on data from hand netting, while data from pan traps were only used to gain information on the potential pool of visitors to experimental plants. Data from pan trap sampling were not used to test for effects of plant nativity on bee community composition due to the close proximity of native and non‐native plots. Bees with uncertain identification were included in analyses of abundances, but not in analyses of bee diversity or bee community composition.

## 3 RESULTS

Over the 2‐year study period, 17 out of the 20 initially seeded pollinator friendly plants came to flower within native plant plots and 18 within non‐native plant plots (Tables 1 and 2). Non‐native plants started flowering about 5 weeks earlier than native plants in both years (Tables 1 and 2). In the second year, many plant species of both plant types started flowering about 2 months earlier and also flowered longer. In both years, both native and non‐native plants were still flowering when observations ceased.

A total of 3,744 bees representing 31 genera and 120 species were recorded in this study. Pan traps sampled 2,036 bees, representing 30 different genera and 107 different species (see Table S1 for complete species list). With hand netting, 1,708 bees were sampled, comprising 25 different genera and 72 different species (Table S1). Some of the bee species were only recorded with one of the two sampling methods: Pan trapping sampled 89% of the total of 120 bees, and 48 species (40%) were sampled exclusively. Hand netting sampled 60% of all species seen in this study, 13 species (11%) exclusively.

**Figure 4:**
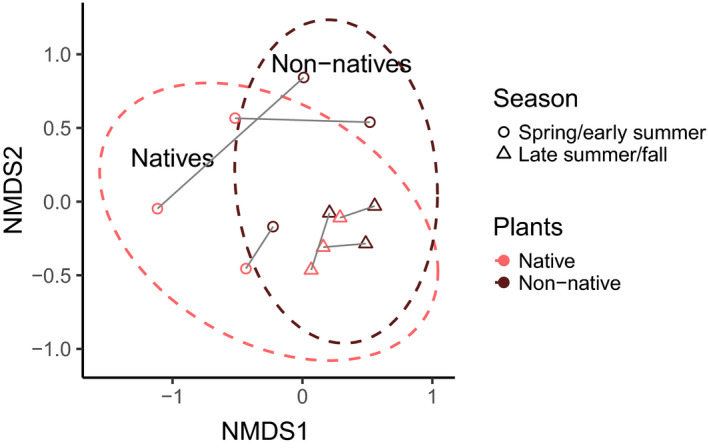
Differences in bee community composition between native plant plots (pink symbols) and non‐native plant plots (dark red symbols) displayed by non‐metric multidimensional scaling (NMDS, stress value = 0.14). Sites are plotted for spring/early summer (circles) and late summer/fall (triangles) separately, resulting in two data points per site. Each symbol represents one site in one season. Corresponding native and non‐native plots of the same site in the same season are connected with gray lines

On native plants, a total of 719 bees of 20 genera and 49 species were hand netted, and a total of 989 bees of 23 genera and 63 species were hand netted on non‐native plants. When comparing only sampling events in which all plots were sampled (once both native and non‐native plants started flowering), we found 718 bees at native and 881 bees at non‐native plots. Bee community compositions differed between native and non‐native plots (PERMANOVA: *p* = a, *df* = 1; Figure 4). Of the total of 72 hand‐netted species, 11 species were captured only within native plots and 23 species only within non‐native plots. Many bee species, such as *Apis mellifera*, *Halictus poeyi/ligatus*, *Bombus bimaculatus,* and *Lasioglossum tegulare*, occurred on both native and non‐native plants, but were more abundant within non‐native plots (e.g., 88 vs 382 visits to native and non‐native plants, respectively, by *A. mellifera*; Table S1). Other bee species, such as *Xylocopa virginica*, *Lasioglossum trigeminum*, and *Augochloropsis metallica_metallica,* were more abundant on native plants (e.g., 228 vs 16 visits to native and non‐native plants, respectively, by *X. virginica*; Table S1). *Apis mellifera* was the only non‐native bee species visiting our experimental plants.

We recorded eight oligolectic bee species (Fowler, 2016) in pan traps, two of which visited our experimental plants. Three females of *Osmia distincta* were caught on the native *Penstemon laevigatus* (Table S1 and Figures S1–S6). *Melissodes desponsus* typically forages on *Cirsium* plants (Fowler, 2016) which were not included in our experiment. Almost all *M. desponsus* recorded were males and captured exclusively on non‐native plants (i.e., on *Cosmos bipinnatus* (7 males, 1 female) and on *Daucus carota* (1 male)) (Table S1 and Figures S1–S6).

**Figure 5:**
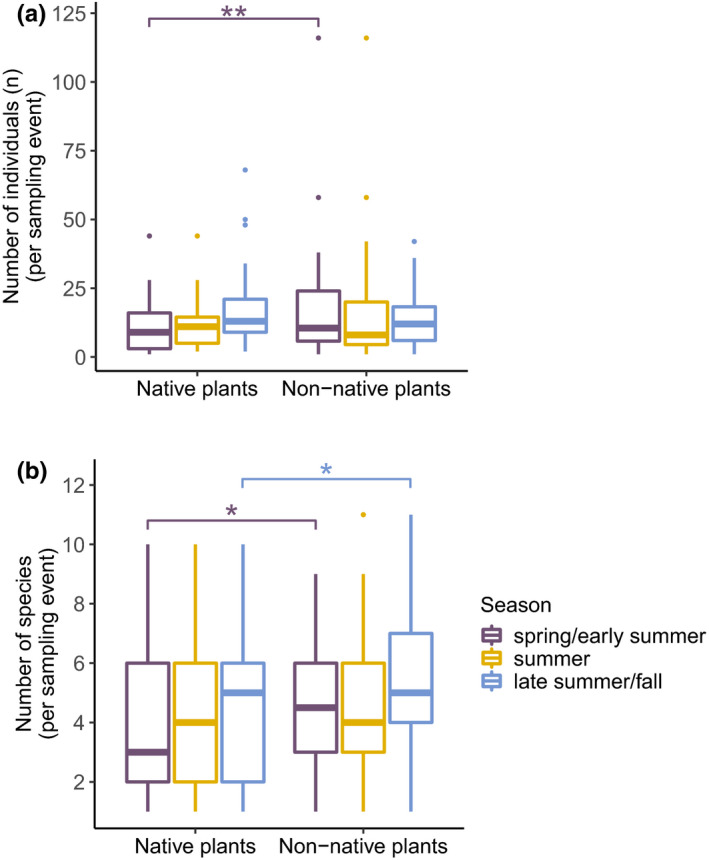
Bee abundance (a) as number of individuals (n) and bee species richness (b) per sampling event at native and non‐native plots by season (data pooled for both observation years). Statistical differences between native versus non‐native plants per season were assessed with LMMs (Table 3) and are indicated here with asterisks when significant: *p < .05, **p < .01. This and the following box plots show the interquartile range (IQR) of data, which extends from the 25th percentile to the 75th percentile. The line within the box indicates the median. The lower whiskers extend to the smallest observation, but not further than 1.5 * IQR from the 25th percentile. The upper whiskers extend to the largest observation, but not further than 1.5 * IQR from the 75th percentile. Outliers are plotted as individual points

Bee abundance and species richness recorded per hand‐netting event differed between native and non‐native plots in some but not all seasons (Figure 5, Table 3). In spring and early summer, both abundance and species richness were significantly lower at native plots than at non‐native plots (Figure 5, Table 3). Species richness was also significantly lower at native plots in late summer and fall (Figure 5, Table 3). These differences of bee abundance and species richness at native and non‐native plots per sampling event also remained when restricting analyses of spring and early summer to sampling events where both plant types were flowering (Table S2, also see 2.3 Statistics).

**Table 3:** Results of the linear mixed‐effect models (LMM) for bee species richness and abundance in the three different seasons with plot type (native/non‐native) as explanatory variable and site and date as random effects

Response variable	*chi^2^*	*df*	*p*	marginal *R^2^*	conditional *R^2^*
Species richness (spring/early summer) Native: 4.3 ± 3.4 Non‐native: 4.7 ± 2.3	3.91	1	< 0.05*	0.07	0.40
Species richness (summer) Native: 4.5 ± 3.0 Non‐native: 4.7 ± 2.4	1.79	1	0.18	0.02	0.30
Species richness (late summer/fall) Native: 4.4 ± 2.6 Non‐native: 5.4 ± 2.5	5.59	1	< 0.05*	0.06	0.33
Abundance (spring/early summer) Native: 12.2 ± 12.4 Non‐native: 20.2 ± 24.9	7.08	1	< 0.01**	0.11	0.52
Abundance (summer) Native: 12 ± 9.0 Non‐native: 16.5 ± 22.2	0.36	1	0.55	< 0.01	0.30
Abundance (late summer/fall) Native: 16.9 ± 15.0 Non‐native: 13.9 ± 10.0	0.16	1	0.69	< 0.01	0.37

Note

The marginal *R^2^*‐value gives the variance explained by the fixed effects and the conditional *R^2^*‐value variance explained by both fixed and random effects. Values of species richness and abundance (by plant type per season) are indicated as mean ± *SD*. Asterisks indicate a significant effect of plant type (in bold): *p < .05, **p < .01.

When we analyzed the subset of data for which floral cover and floral richness were available (year 2017), we found that bee abundance and bee species richness were best explained by floral cover and increased with increasing proportions of floral cover (Table S3). Additionally, bee abundance increased with plant diversity in the early and late season as did bee species richness in the early season (Table S3). Moreover, in the late season, more bee species visited non‐native than native plots (Table S3). In spring and early summer of 2017, floral richness was twice as high in non‐native (mean ± *SD*: 7 ± 2) than native (3 ± 2) plots while floral cover was similar (native: 15.9% ± 19.0%; non‐native: 17.0% ± 14.4%). In summer of 2017, floral cover was highly variable and floral richness similar across plot types (floral cover, native: 35.3% ± 20.7%; non‐native: 24.3% ± 12.1%; floral richness, native: 6 ± 1; non‐native: 7 ± 2), and in late summer and fall, floral cover and richness were higher in native than non‐native plots (floral cover, native: 40.0% ± 20.4%; non‐native: 21.2% ± 11.8%; floral richness, native: 7 ± 1; non‐native: 5 ± 2 (mean ± *SD*)).

The specialization (H2′) of the recorded plant–bee networks also differed between plots and seasons (Table 4, Figures S1–S6). Native plants were part of more specialized networks than non‐native plants, and early season networks were more specialized than late season networks (Table 4). These trends also remained when removing honeybees (*A. mellifera*) from networks (Table S4, also see 2.3 Statistics). Early season networks included fewer plant and bee species (Figures S1–S6).

**Table 4:** Results of the linear mixed‐effect models (LMM) for the plant–bee network specialization (H2′) and for individual specialization (*d*′) of the five most abundant bee species with plant nativity and season as explanatory variables and site as random factor; and results (direction of seasonal difference and *p*‐values) of the Tukey post hoc tests on differences between seasons

	Plant nativity			Season			Tukey's post hoc for season			
	*chi^2^*	*df*	*p*	*chi^2^*	*df*	*p*	Direction	*p*	Marginal *R^2^*	Conditional *R^2^*
**H2′** Native: 0.64 ± 0.11 Non‐native: 0.44 ± 0.18 Early season: 0.67 ± 0.11 Midseason: 0.51 ± 0.13 Late season: 0.45 ± 0.21	12.19	1	<0.001***	11.03	2	<0.01**	early > late	<0.01**	0.58	0.58
***d*′** (*Apis mellifera*): 0.74 ± 0.21	2.00	1	0.16	35.32	2	<0.001***	early > late mid > late	<0.001*** <0.001***	0.56	0.77
***d*′** (*Bombus impatiens*): 0.60 ± 0.15	8.30	1	<0.01**	1.09	1	0.30			0.29	0.66
***d*′** (*Halictus poeyi/ligatus*): 0.66 ± 0.15	1.33	1	0.25	14.19	2	0.12			0.25	0.25
***d*′** (*Lasioglossum pilosum*): 0.53 ± 0.13	4.06	1	<0.05*	1.49	2	0.48			0.38	0.38
***d*′** (*Xylocopa virginica*): 0.83 ± 0.16	33.49	1	<0.001***	0.04	1	0.85			0.81	0.81

Note

Values of *d*′ and H2′ (by plant type and by season) are indicated as mean ± *SD*. H2′ values of each network are included in Figures S1–S6. The marginal *R^2^*‐value gives the variance explained by the fixed effects and the conditional *R^2^*‐value variance explained by both fixed and random effects. Species specialization of *Bombus impatiens* and *Xylocopa virginica* only included data of two seasons, summer and late summer to fall, because these species were not present in spring to early summer. Seasons are abbreviated as follows: early = spring to early summer; mid = summer; and late = late summer to fall. Asterisks indicate significant effects (in bold): *p < .05, **p < .01, ***p < .001.

**Figure 6:**
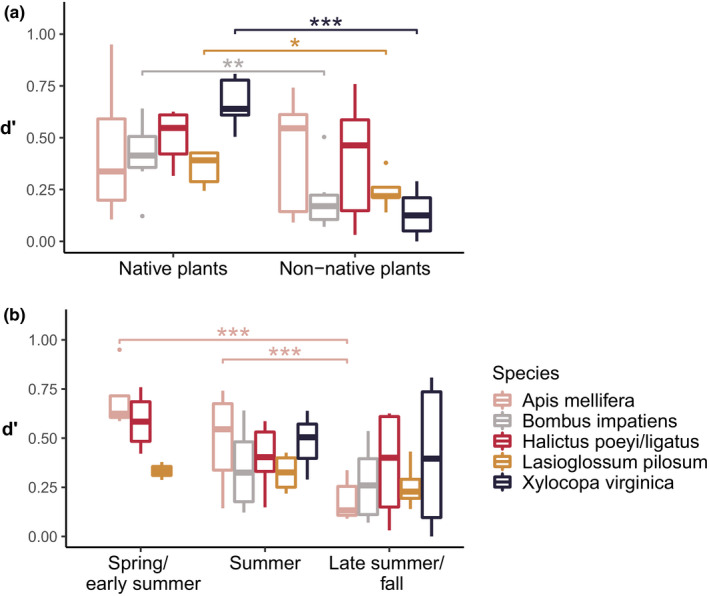
Species specificity (*d*′) of the five most abundant bee species shown for (a) native and non‐native plantings and for (b) season. The *d*′ index provides information on the degree of specialization of individual species and ranges from 0 (not specialized) to 1 (highly specialized). It is based on interaction links between bee species and plant species, but also takes into account the dominance of each linked plant species in the interaction network. Thus, bee species visiting the most dominant plant species are considered less specialized than bee species visiting plants that are rarely visited by other bee species. Statistical differences between plant types and seasons per bee species were assessed with LMMs (Table 4) and are indicated here with asterisks when significant: *p < .05, **p < .01, ***p < .001

The five most abundant bee species across both native and non‐native plant plots were *A. mellifera*, *H. poeyi/ligatus*, *X. virginica*, *B. impatiens*, and *Lasioglossum pilosum*, in order of decreasing abundance (Table S1). *Xylocopa virginica*, *B. impatiens*, and *L. pilosum* showed a more specialized foraging behavior at native than at non‐native plants (Figure 6 and Table 4). *Apis mellifera* became less specialized over the entire flowering season with the highest specialization early in the year and lowest specialization later in the year (Figure 6 and Table 4). The other species' specificity remained similar across seasons, but *X*. *virginica* and *B. impatiens* were absent in the early season (Figure 6 and Table 4).

## 4 DISCUSSION

Our study showed that a seed mix of non‐native pollinator friendly plants was well accepted and frequented by a diverse bee community. Depending on the season, non‐native plants attracted either similar or higher numbers of individuals and species compared to native plants. Particularly in the early and late season, many bees chose non‐native over native plants, while no differences were found in the middle of summer. Generally, bee visitation increased with increasing floral resource coverage, which has also been shown by previous studies (Banaszak, 1996; Batáry et al., 2010; Baude et al., 2016) and may explain some of the observed differences between native and non‐native plots. For spring and early summer, native plants were still scarce which likely contributed to the smaller number of bees sampled. During summer and fall, when numbers of flowers were similar across plots, non‐native plants still attracted more bees. One possible explanation for this difference is that late flowering non‐native plants offered higher quantities of nutritionally more attractive floral resources. Alternatively, non‐native plant flowers may have had more attractive visual or olfactory cues than native plants.

Previous studies comparing the attractiveness of native and non‐native plants found inconsistent results. Notably, none of these studies strictly focused on pollinator friendly plants or differentiated between seasons (e.g., Lopezaraiza‐Mikel et al., 2007; Morales & Aizen, 2006; Morandin & Kremen, 2013; Williams et al., 2011), which may explain why our study found non‐native plants to be, depending on the season, similarly or more attractive to bees compared to native plants.

Native and non‐native plant communities also differed in the composition of bees they attracted. For example, honeybees (*A. mellifera*) or the sweat bee *L. tegulare* were more abundant on non‐native plants than on native plants, while other polylectic bees, such as *X. virginica* or *L. trigeminum*, were more abundant on native than on non‐native plants. This result supports previous findings showing bee species‐specific responses to non‐native plants (Pardee & Philpott, 2014; Schweiger et al., 2010; Urbanowicz et al., 2020), which may be due to species‐specific nutritional requirements (Leonhardt & Blüthgen, 2012; Nicolson, 2011; Vaudo et al., 2016), previous experiences (Harmon‐Threatt & Kremen, 2015; Vaudo et al., 2015), or competition (Somme et al., 2015; Wilms et al., 1996).

Additionally, plant–bee networks differed between native and non‐native plants. As expected, networks associated with native plants were more specialized than networks associated with non‐native plants. Native plants share a longer evolutionary history of interactions with native bees than non‐native plants, which likely increased the chance of more specialized interactions evolving (Fenster et al., 2004). Other studies found that non‐native plants were predominantly visited by more generalized insects (Aizen et al., 2008; Lopezaraiza‐Mikel et al., 2007; Memmott & Waser, 2002; Olesen et al., 2002; Schweiger et al., 2010; Stout & Morales, 2009). Non‐native plants therefore appear to favor generalist (or polylectic) over specialist (or oligolectic) bees. This raises concerns about the use of non‐native plants in seed mixes aimed at supporting bees and other organisms (Bartomeus et al., 2008; Lopezaraiza‐Mikel et al., 2007; Winfree et al., 2011). In our study, we recorded only two oligolectic bee species visiting flowers, preventing more robust inferences on disproportional effects on specialists. In the mid‐Atlantic region, oligolectic bee activity peaks in summer (with up to 57 spp. active in June), followed by spring and fall (Fowler, 2016). Our survey might have missed some of the early oligolectic bee species due to the late start of flowering of our experimental plants, especially at native plots. Although oligolectic bees clearly depend on their native host plants for pollen collection, they appear to use non‐native plants for nectar collection or as resting areas, as suggested by the occurrence of *M. desponsus* males on non‐native plants.

The occurrence of more specialists in a plant–bee network can be one reason for an increased network specialization. Another reason can be more specialized interactions of generalist species. In our study, most bee species were generalists. Although generalist bees typically feed on a broad range of floral resources, their foraging patterns and thus their level of specialization can vary with the spectrum of plants available (Cook et al., 2003; Somme et al., 2015; Vaudo et al., 2016). For example, three out of the five most abundant generalist species in our study showed more specialized foraging when feeding on native plants. While we did find that non‐native networks were comprised of slightly more plant species than native networks, this difference should not have affected network or species specialization, as both *d*′ and H2′ are robust against variations in network size, shape, and sampling intensity (Blüthgen et al., 2006). One reason for a more specialized foraging behavior of generalists on native plants could be overall stronger differences in chemical profiles between native plants, with specific plant species better meeting the nutritional requirements of specific bee species. Native generalist bees could also have evolved a higher degree of specialization on certain native plant species, through adapting to their chemical repertoire.

Network specialization also changed over the seasons for interactions involving both native and non‐native plants. Specialization was highest in the early season and decreased toward fall. The higher degree of specialization coincided with a lower number of plant species as part of the networks in spring, which is not uncommon for grasslands (Mallinger et al., 2016). The drop in network specialization may be a consequence of an increase in relative abundance of very generalist generalists toward the end of the flowering season (e.g., honeybees and bumblebees). These bees can forage on a broad spectrum of plant species and tended to also decrease their level of specialization toward the end of the flowering season. Unfortunately, we are not aware of other studies which have assessed changes in network and species specialization over the flowering season and can therefore not relate our findings to other studies.

The flowering periods of our plants changed from one year to the next. In the second year, many plants started flowering earlier and flowered for longer periods than in the first year. These changes were likely due to the early stage of the (small scale) meadow established in the first year. Overall, our survey focused on bee visitation of meadows in establishment as opposed to long‐established pollinator friendly vegetation. Lee‐Mäder et al. (2013) indicated that regular flowering of (perennial) wildflower meadows does usually not begin before the third year after establishment. Both native and non‐native plantings had few plant species in the early establishment phase. However, the scarcity of flowers in spring was more pronounced for native than non‐native plots in both years, which likely explains at least part of the differences in bee abundance observed for the spring/early summer period. This agrees with Mallinger et al. (2016) who pointed out that floral resources are often scarce in natural grasslands in spring and that bees therefore rely on additional floral resources provided by woodlands or anthropogenically managed habitats. Where woodlands are not available, non‐native plants as part of pollinator friendly meadows may provide valuable resources for the local bee community at times when native plants are still scarce or absent. Their inclusion in pollinator friendly seed mixes should nevertheless be treated with extreme caution, because, even though they may benefit pollinator communities, non‐native plants, such as invasive plants, may exert unpredictable and potentially detrimental pressures on native animal and plant communities (Chrobock, et al., 2013).

To conclude, our study suggests that non‐native plants can complement native pollinator friendly plantings, because they are visited by a broad spectrum of bees and buffer gaps in grassland native plant flowering times, particularly in early spring. However, non‐native plants also alter the composition of plant communities, may not support as many specialist bees, and appear to affect individual and network specialization of bee communities with unknown consequences for plants and bees. Given the already severe alteration of our ecosystems as a result of anthropogenic activities worldwide, the use of selected non‐native plants in meadows or flower strips along crop fields as food resources for bees and other pollinators can be considered a pragmatic possibility to partially compensate for the scarcity of natural habitats and native plants in landscapes heavily dominated by humans. We should, however, make sure to prevent the spread of non‐native plant species to (semi‐)natural areas, where they may disturb established natural plant and bee communities with unknown consequences for these ecosystems. We therefore suggest that non‐native plants should only be included, with caution, in pollinator friendly plant mixes used in human‐dominated landscapes and only when complementary to native pollinator friendly plants. Furthermore, our results highlight the importance of analyzing entire flowering periods instead of focusing solely on specific seasons (e.g., summer) and of taking into account phenological matching for network analyses (see also Kantsa et al. (2017)). More research that experimentally compares native versus non‐native pollinator friendly plant mixes in different regions of the northern and southern hemisphere, at different scales and with other plant species would be helpful in order to best support local bee communities and to globally improve conservation strategies for bees.

## CONFLICT OF INTEREST

The authors declare no competing interests.

## AUTHOR CONTRIBUTIONS

**Nicola Seitz:** Conceptualization (equal); data curation (lead); formal analysis (lead); investigation (lead); methodology (equal); project administration (lead); visualization (lead); writing‐original draft (lead); writing–review and editing (equal). **Dennis vanEngelsdorp:** Conceptualization (equal); Resources (equal); supervision (equal); writing–review and editing (equal). **Sara Diana Leonhardt:** Conceptualization (equal); methodology (equal); resources (equal); supervision (equal); writing–review and editing (equal).

## Supporting information

Appendix S1

## Data Availability

All data used in analyses can be found in Dryad at https://doi.org/10.5061/dryad.pzgmsbcj8.
