# Supplementary material for: Are native and non‐native pollinator friendly plants equally valuable for native wild bee communities?
Source: Ecol Evol. 2020 Oct 13;10(23):12838–50. doi: 10.1002/ece3.6826 (PMC7713930; doi:10.1002/ece3.6826)
Supplement: Supplementary file 1 — Appendix S1 [file ECE3-10-12838-s001.docx]

**Appendix Table S1.** List of all bee species sampled by sampling method including number of individuals. Oligolectic foraging is indicated with an x, based on Fowler (2016).

|  | **Number of individuals (n)** | | | **Oligolectic bees (host plant genus; according to Fowler (2016))** |
| --- | --- | --- | --- | --- |
| **Species** | **handnetting native plots** | **handnetting non-native plots** | **pan traps** |  |
| Agapostemon |  | 1 |  |  |
| Agapostemon sericeus |  |  | 1 |  |
| Agapostemon splendens | 3 | 18 | 29 |  |
| Agapostemon texanus | 3 | 7 | 118 |  |
| Agapostemon virescens | 5 | 5 | 42 |  |
| Andrena (Trachandrena) |  | 1 | 3 |  |
| Andrena atlantica |  | 2 |  |  |
| Andrena banksi |  |  | 2 |  |
| Andrena barbara |  |  | 3 |  |
| Andrena carlini |  |  | 3 |  |
| Andrena cressonii |  |  | 1 |  |
| Andrena erigeniae |  |  | 1 | x (Claytonia) |
| Andrena imitatrix/morrisonella |  |  | 1 |  |
| Andrena macra |  |  | 2 |  |
| Andrena miserabilis |  |  | 4 |  |
| Andrena nasonii |  |  | 16 |  |
| Andrena perplexa |  | 1 | 6 |  |
| Andrena vicina |  |  | 1 |  |
| Andrena violae |  |  | 2 | x (Viola) |
| Andrena wilkella |  | 13 | 4 |  |
| Anthidiellum notatum |  | 4 | 2 |  |
| Anthidium manicatum |  |  | 2 |  |
| Anthidium oblongatum |  |  | 1 |  |
| Apis mellifera | 3 | 23 | 84 |  |
| Augochlora pura | 2 | 3 | 45 |  |
| Augochlorella aurata | 11 | 15 | 216 |  |
| Augochloropsis metallica_fulgida | 1 | 2 |  |  |
| Augochloropsis metallica_metallica | 6 | 2 | 6 |  |
| Bombus |  | 4 |  |  |
| Bombus bimaculatus | 8 | 22 | 8 |  |
| Bombus citrinus | 1 | 10 |  |  |
| Bombus fervidus |  |  | 3 |  |
| Bombus fervidus/pensylvanicus |  | 2 | 6 |  |
| Bombus griseocollis | 3 | 5 | 2 |  |
| Bombus impatiens | 31 | 41 | 28 |  |
| Bombus pensylvanicus |  | 3 | 1 |  |
| Calliopsis andreniformis | 1 | 2 | 55 |  |
| Ceratina |  | 4 | 3 |  |
| Ceratina calcarata | 15 | 16 | 22 |  |
| Ceratina dupla | 1 | 1 | 4 |  |
| Ceratina mikmaqi | 1 |  | 6 |  |
| Ceratina strenua | 1 | 2 | 9 |  |
| Coelioxys octodentata | 1 | 1 | 1 |  |
| Coelioxys sayi |  | 1 |  |  |
| Eucera hamata |  |  | 13 |  |
| Halictus confusus | 2 | 1 | 11 |  |
| Halictus parallelus |  | 4 | 1 |  |
| Halictus poeyi/ligatus | 173 | 254 | 198 |  |
| Halictus rubicundus |  |  | 4 |  |
| Heriades carinata |  | 1 |  |  |
| Hoplitis pilosifrons |  |  | 2 |  |
| Hoplitis producta |  |  | 2 |  |
| Hoplitis trunactum | 2 |  |  |  |
| Hoplitis truncata |  |  | 1 |  |
| Hylaeus affinis/modestus | 1 | 4 | 8 |  |
| Hylaeus modestus |  | 2 | 1 |  |
| large green bee | 6 |  |  |  |
| Lasioglossum | 7 | 6 | 40 |  |
| Lasioglossum abanci |  |  | 2 |  |
| Lasioglossum admirandum |  |  | 7 |  |
| Lasioglossum bruneri |  | 2 | 26 |  |
| Lasioglossum callidum | 4 | 2 | 18 |  |
| Lasioglossum coeruleum |  | 1 |  |  |
| Lasioglossum coreopsis | 10 | 11 | 39 |  |
| Lasioglossum coriaceum | 3 |  | 33 |  |
| Lasioglossum cressonii |  | 1 | 13 |  |
| Lasioglossum ephialtum |  |  | 6 |  |
| Lasioglossum floridanum |  |  | 14 |  |
| Lasioglossum fuscipenne | 3 | 1 | 1 |  |
| Lasioglossum gotham |  |  | 1 |  |
| Lasioglossum hitchensi | 8 | 7 | 53 |  |
| Lasioglossum imitatum | 1 | 1 |  |  |
| Lasioglossum leucocomum |  |  | 2 |  |
| Lasioglossum nelumbonis |  |  | 1 |  |
| Lasioglossum oblongum | 1 |  | 5 |  |
| Lasioglossum pectorale | 1 | 1 | 3 |  |
| Lasioglossum pilosum | 19 | 19 | 232 |  |
| Lasioglossum subviridatum | 1 | 4 | 36 |  |
| Lasioglossum tegulare | 4 | 9 | 104 |  |
| Lasioglossum trigeminum | 11 | 2 | 82 |  |
| Lasioglossum versatum | 5 | 3 | 39 |  |
| Lasioglossum vierecki | 2 | 2 | 9 |  |
| Lasioglossum weemsi |  | 2 | 5 |  |
| Megachile brevis |  | 1 | 22 |  |
| Megachile exilis | 3 | 4 | 2 |  |
| Megachile gemula |  |  | 1 |  |
| Megachile georgica | 1 |  | 1 |  |
| Megachile inimica | 1 | 3 | 1 |  |
| Megachile mendica | 6 | 8 | 8 |  |
| Megachile montivaga |  |  | 1 |  |
| Megachile petulans |  | 1 |  |  |
| Megachile texana | 1 |  |  |  |
| Melissodes | 1 |  | 2 |  |
| Melissodes bimaculatus |  | 5 | 72 |  |
| Melissodes comptoides |  |  | 8 |  |
| Melissodes denticulata |  |  | 3 | x (Vernonia) |
| Melissodes desponsus |  | 9 | 1 | x (Cirsium) |
| Melissodes subillatus | 9 |  |  |  |
| Melissodes trinodis | 2 |  | 1 |  |
| Melitoma taurea |  |  | 2 | x (Ipomoea) |
| NA | 3 | 3 | 11 |  |
| Nomada |  |  | 2 |  |
| Nomada articulata |  | 8 | 27 |  |
| Nomada australis |  | 4 | 36 |  |
| Nomada bidentate_group |  |  | 4 |  |
| Nomada denticulata |  |  | 3 |  |
| Nomada imbricata |  |  | 1 |  |
| Nomada luteoloides |  |  | 1 |  |
| Nomada maculata |  |  | 1 |  |
| Nomada parva | 1 | 1 | 2 |  |
| Nomada pygmaea |  |  | 4 |  |
| Nomada texana | 1 |  |  |  |
| Nomada vegana |  | 2 |  |  |
| Osmia atriventris |  | 2 | 8 |  |
| Osmia bucephala |  |  | 8 |  |
| Osmia collinsiae |  |  | 4 |  |
| Osmia distincta | 3 |  | 2 | x (Penstemon) |
| Osmia georgica |  | 1 | 2 |  |
| Osmia pumila |  | 2 | 22 |  |
| Osmia sandhouseae |  |  | 1 |  |
| Osmia subfasciata |  |  | 3 |  |
| Osmia taurus |  |  | 1 |  |
| Peponapis pruinosa |  |  | 1 | x (Cucurbita) |
| Pseudopanurgus near_labrosiformis |  |  | 4 |  |
| Ptilothrix bombiformis |  |  | 1 | x (Hibiscus) |
| small dark bee | 9 | 4 |  |  |
| small green bee | 2 |  |  |  |
| Sphecodes |  | 2 | 1 |  |
| Stelis louisae | 1 |  | 1 |  |
| Svastra obliqua |  | 2 | 1 |  |
| Triepeolus lunatus | 1 | 1 | 2 |  |
| Triepeolus remigatus |  |  | 1 |  |
| Xylocopa virginica | 11 | 4 | 1 |  |

**Appendix Table S2.** Results of the linear mixed effect models (LMM) for bee species richness and abundance in spring with plot type (native/non-native) as explanatory variable and site and date as random effects based only on sampling events where both native and non-native plots were in flower/sampled. The marginal *R^2^*-value gives the variance explained by the fixed effects and the conditional *R^2^*-value variance explained by both fixed and random effects. Values of species richness and abundance are indicated as mean ± sd. Asterisks indicate a significant effect of plant type (in bold): ** *p* < 0.01; borderline significance: . p < 0.1.

| **Response variable** | ***chi^2^*** | **df** | ***p*** | **marginal *R****^2^* | **conditional *R****^2^* |
| --- | --- | --- | --- | --- | --- |
| Species richness (spring/early summer)  Native: 4.6 ± 3.4  Non-native: 5.7 ± 2.4 | 2.94 | 1 | 0.087 . | 0.07 | 0.42 |
| Abundance (spring/early summer)  Native: 13.1 ± 12.5  Non-native: 31.5 ± 31.2 | 6.77 | 1 | **< 0.01**** | 0.14 | 0.53 |

**Appendix Table S3.** Results of the linear mixed effect models (LMM) for bee species richness and abundance in the three different seasons of 2017 only, with plot type (native/non-native), floral cover (proportion of ground with flowering vegetation) and floral species richness as explanatory variables and site and date as random effects. Data of bee abundance was log transformed in order to obtain normal distribution. The marginal *R^2^*-value gives the variance explained by the fixed effects and the conditional *R^2^*-value variance explained by both fixed and random effects. Values of species richness and abundance (by plant type per season) are indicated as mean ± sd. Asterisks indicate a significant effect of plant type (in bold): * *p* < 0.05, ** *p* < 0.01, *** *p* < 0.001.

|  | **Plant nativity** | | | **Floral cover** | | | **Floral richness** | | |  |  |
| --- | --- | --- | --- | --- | --- | --- | --- | --- | --- | --- | --- |
|  | ***chi^2^*** | **df** | ***p*** | ***chi^2^*** | **df** | ***p*** | ***chi^2^*** | **df** | ***p*** | **marginal *R^2^*** | **conditional *R^2^*** |
| Species richness (spring/early summer 2017)  Native: 5.1 ± 3.5  Non-native: 5.6 ± 2.2 | 1.61 | 1 | 0.20 | 8.94 | 1 | **< 0.01**** | 3.96 | 1 | **< 0.05*** | 0.50 | 0.55 |
| Species richness  (summer 2017)  Native: 5.4 ± 3.0  Non-native: 5.5 ± 2.5 | 1.16 | 1 | 0.28 | 6.48 | 1 | **< 0.05*** | 0.07 | 1 | 0.80 | 0.16 | 0.27 |
| Species richness  (late summer/fall 2017)  Native: 5.2 ± 2.5  Non-native: 5.8 ± 2.8 | 9.01 | 1 | **< 0.01**** | 9.57 | 1 | **< 0.01**** | 2.86 | 1 | 0.09 | 0.23 | 0.41 |
| Abundance  (spring/early summer 2017)  Native: 14.0 ± 13.7  Non-native: 26.9 ± 28.1 | 0.04 | 1 | 0.85 | 15.14 | 1 | **< 0.001***** | 6.03 | 1 | **< 0.05*** | 0.66 | 0.66 |
| Abundance  (summer 2017)  Native: 13.8 ± 10.3  Non-native: 22.2 ± 27.0 | 3.21 | 1 | 0.07 | 8.07 | 1 | **< 0.01**** | < 0.01 | 1 | 0.95 | 0.18 | 0.48 |
| Abundance  (late summer/fall 2017)  Native: 20.4 ± 17.4  Non-native: 14.3 ± 11.5 | 1.61 | 1 | 0.20 | 3.95 | 1 | **< 0.05*** | 5.53 | 1 | **< 0.05*** | 0.15 | 0.58 |

**Appendix Table S4.** Results of the linear mixed effect models (LMM) for plant-bee network specialization (H2’) based on data excluding honey bee visitation, with plant nativity and season as explanatory variables and site as random factor; and results (direction of seasonal difference and *p*-values) of the Tukey post hoc tests on differences between seasons. Values of H2’ (by plant type and by season) are indicated as mean ± sd. The marginal *R^2^*-value gives the variance explained by the fixed effects and the conditional *R^2^*-value variance explained by both fixed and random effects. Seasons are abbreviated as follows: early = spring to early summer; mid = summer; and late = late summer to fall. Asterisks indicate significant effects (in bold): ** *p* < 0.01, *** *p* < 0.001.

|  | **Plant nativity** | | |  | **Season** | | |  | **Tukey post hoc**  **for season** | | | |  |  | |  | |
| --- | --- | --- | --- | --- | --- | --- | --- | --- | --- | --- | --- | --- | --- | --- | --- | --- | --- |
|  | ***chi^2^*** | **df** | ***p*** |  | ***chi^2^*** | **df** | ***p*** |  | **direction** | ***p*** |  | **marginal *R^2^*** | | | **conditional *R^2^*** | |  |
| **H2’**  Native: 0.61 ±0.14  Non-native: 0.40 ±0.14  Early season: 0.61 ±0.14  Mid-season: 0.42 ±0.11  Late season: 0.48 ±0.21 | 13.80 | 1 | **< 0.001***** |  | 8.27 | 2 | **< 0.01**** |  | early > mid | **< 0.01**** |  | 0.56 | | | 0.56 | |  |

**Appendix Figure S1.** Plant-bee networks at native plots of site A, (top) in spring/early summer (H2’ = 0.74); (middle) in summer (H2’ = 0.50); and (bottom) in late summer/fall (H2’ = 0.59). The H2’ value gives the degree of network specialization and ranges from 0 (no specialization) to 1 (highest specialization). Bee species are indicated by black bars in the upper network levels. Thickness of bars relates to the bee species’ abundance within the network. Plant species are indicated by black bars in the lower network levels. Here, thickness of bars relates to the number of visits a plant species received. Grey lines between bee and plant species show interaction links. Thickness of these connecting lines relates to the frequency of visits.

**Appendix Figure S2.** Plant-bee networks at native plots of site B, (top) in spring/early summer (H2’ = 0.71); (middle) in summer (H2’ = 0.58); and (bottom) in late summer/fall (H2’ = 0.61). The H2’ value gives the degree of network specialization and ranges from 0 (no specialization) to 1 (highest specialization). Bee species are indicated by black bars in the upper network levels. Thickness of bars relates to the bee species’ abundance within the network. Plant species are indicated by black bars in the lower network levels. Here, thickness of bars relates to the number of visits a plant species received. Grey lines between bee and plant species show interaction links. Thickness of these connecting lines relates to the frequency of visits.

**Appendix Figure S3.** Plant-bee networks at native plots of site C, (top) in spring/early summer (H2’ = 0.82); (middle) in summer (H2’ = 0.5); and (bottom) in late summer/fall (H2’ = 0.68). The H2’ value gives the degree of network specialization and ranges from 0 (no specialization) to 1 (highest specialization). Bee species are indicated by black bars in the upper network levels. Thickness of bars relates to the bee species’ abundance within the network. Plant species are indicated by black bars in the lower network levels. Here, thickness of bars relates to the number of visits a plant species received. Grey lines between bee and plant species show interaction links. Thickness of these connecting lines relates to the frequency of visits.

**Appendix Figure S4.** Plant-bee networks at non-native plots of site A, (top) in spring/early summer (H2’ = 0.61); (middle) in summer (H2’ = 0.57); and (bottom) in late summer/fall (H2’ = 0.27). The H2’ value gives the degree of network specialization and ranges from 0 (no specialization) to 1 (highest specialization). Bee species are indicated by black bars in the upper network levels. Thickness of bars relates to the bee species’ abundance within the network. Plant species are indicated by black bars in the lower network levels. Here, thickness of bars relates to the number of visits a plant species received. Grey lines between bee and plant species show interaction links. Thickness of these connecting lines relates to the frequency of visits.

**Appendix Figure S5.** Plant-bee networks at non-native plots of site B, (top) in spring/early summer (H2’ = 0.59); (middle) in summer (H2’ = 0.64); and (bottom) in late summer/fall (H2’ = 0.34). The H2’ value gives the degree of network specialization and ranges from 0 (no specialization) to 1 (highest specialization). Bee species are indicated by black bars in the upper network levels. Thickness of bars relates to the bee species’ abundance within the network. Plant species are indicated by black bars in the lower network levels. Here, thickness of bars relates to the number of visits a plant species received. Grey lines between bee and plant species show interaction links. Thickness of these connecting lines relates to the frequency of visits.

**Appendix Figure S6.** Plant-bee networks at non-native plots of site C, (top) in spring/early summer (H2’ = 0.51); (middle) in summer (H2’ = 0.28); and (bottom) in late summer/fall (H2’ = 0.18). The H2’ value states network specialization and ranges from 0 (no specialization) to 1 (highest specialization). Bee species are indicated by black bars in the upper network levels. Thickness of bars relates to the bee species’ abundance within the network. Plant species are indicated by black bars in the lower network levels. Here, thickness of bars relates to the number of visits a plant species received. Grey lines between bee and plant species show interaction links. Thickness of these connecting lines relates to the frequency of visits.
